# Performance of Generative Pre-trained Transformer (GPT)-4 and Gemini Advanced on the First-Class Radiation Protection Supervisor Examination in Japan

**DOI:** 10.7759/cureus.70614

**Published:** 2024-10-01

**Authors:** Hiroki Goto, Yoshioki Shiraishi, Seiji Okada

**Affiliations:** 1 Radioisotope and Tumor Pathobiology, Institute of Resource Development and Analysis, Kumamoto University, Kumamoto, JPN; 2 Radioisotope Center, Institute of Resource Development and Analysis, Kumamoto University, Kumamoto, JPN; 3 Hematopoiesis, Joint Research Center for Human Retrovirus Infection, Kumamoto University, Kumamoto, JPN

**Keywords:** artificial intelligence (ai), gemini advanced, generative pre-trained transformer-4, large language model, radiation safety and protection

## Abstract

​Purpose

The purpose of this study was to evaluate the capabilities of large language models (LLMs) in understanding radiation safety and protection. We assessed the performance of generative pe-trained transformer (GPT)-4 (OpenAI, USA) and Gemini Advanced (Google DeepMind, London) using questions from the First-Class Radiation Protection Supervisor Examination in Japan.

Methods

The study involved GPT-4 and Gemini Advanced answering questions from the 68th First-Class Radiation Protection Supervisor Examination in Japan. The number of correct and incorrect answers based on the subject, the presence or absence of calculation, the passage length, and the format (textual or graphical questions) were analyzed in this study. Comparisons of the results between GPT-4 and Gemini Advanced were performed.

Results

The overall accuracy rates of GPT-4 and Gemini Advanced were 71.0% and 65.3%, respectively. A significant difference was observed in the subject (P < 0.0001 in GPT-4 and P = 0.0127 in Gemini Advanced). The accuracy rate of laws and regulations was lower than in the other subjects. There was no significant difference in the presence or absence of calculation or the passage length. The performance of both LLMs was significantly better in textual questions than in graphical questions (P = 0.0003 in GPT-4 and P < 0.0001 in Gemini Advanced). The performance of the two LLMs did not differ significantly based on the subject, the presence or absence of calculation, the passage length, or the format.

Conclusions

GPT-4 and Gemini Advanced demonstrated sufficient understanding of physics, chemistry, biology, and practical operations to meet the passing standard for the average score. However, in laws and regulations, their performance was insufficient, possibly due to frequent revisions and the complexity of detailed regulations, and further machine learning is required.

## Introduction

The development of artificial intelligence (AI) is having a significant impact across a wide range of fields including education and healthcare [1,2]. In healthcare, AI is being increasingly applied to diagnose diseases through medical imaging and to analyze medical records with the help of natural language processing (NLP) tools [3,4]. AI chatbots are programs that use AI to automatically engage in conversations with humans. Lately, AI chatbots have primarily utilized NLP technology to understand human language and generate appropriate responses [5]. Generative pre-trained transformer (GPT)-4, developed by OpenAI (USA), and Gemini Advanced, created by Google DeepMind (London), are advanced AI chatbots designed to engage in natural conversations. They are large language models (LLMs) capable of understanding and generating text, handling various tasks such as answering questions, providing information, and producing creative content.

ChatGPT and GPT-4 were able to answer questions correctly on the United States Medical Licensing Examination (USMLE) [5,6] and the Japanese National Medical Licensing Examination [7,8] at a level sufficient to pass, despite not being trained in a specific discipline. As a result, their applications in healthcare education are anticipated [9]. ChatGPT has sparked interest in the potential uses of LLMs in radiology [10-12]. Although LLMs show promising applications, their use in the training and support of more specialized healthcare fields, such as radiation safety and protection, remains largely unexplored.

A Radiation Protection Supervisor is responsible for safely managing radiation and radioactive materials at various locations such as research institutes, hospitals, and nuclear facilities, overseeing and guiding efforts to prevent radiation exposure to workers and the environment. In Japan, the Nuclear Regulation Authority (NRA) administers the Radiation Protection Supervisor Examination and issues licenses to those who pass. The NRA conducts three types of Radiation Protection Supervisor exams: first-class, second-class, and third-class Radiation Protection Supervisor. The First-Class Supervisor can handle a broader range of high-energy radiation and larger quantities of radioactive materials, while the Second or Third-Class Supervisor is limited to lower levels of radiation and smaller-scale operations.

According to the foregoing, while AI chatbots are expected to be useful tools that can contribute to healthcare education and practice, their usefulness in the field of radiation safety and protection, which is also a more specialized field, has been limitedly explored. In this study, we assessed the ability of GPT-4 and Gemini Advanced to understand radiation safety and protection through the First-Class Radiation Protection Supervisor Examination in Japan.

## Materials and methods

GPT-4 and Gemini Advanced

OpenAI and Google developed LLMs, offering highly advanced capabilities in NLP and multimodal tasks, such as handling text and images [13]. In OpenAI's GPT series, GPT-4 was released in March 2023. GPT-4V (the visual version of GPT-4) was released in September 2023 as a part of the GPT-4 model, adding vision capabilities to the existing text-based functionality. As of November 2023, it was fully integrated into the general use of GPT-4, with no longer a separate option for vision, becoming a standard feature of the model. GPT-4 is primarily available through OpenAI's paid service, ChatGPT Plus, which costs $20 per month. This plan gives users access to GPT-4's advanced capabilities, offering improved text generation and performance compared with GPT-3.5.

Google has also been continuously evolving its LLMs, starting from Bard, then to Gemini, and now to Gemini Advanced. Google's Bard transitioned to Gemini in December 2023, with the release of Gemini 1.0. Gemini Advanced is a paid version (monthly subscription of $20) of Gemini, a generative AI service offered by Google, and is powered by Google's state-of-the-art AI model, Gemini Ultra 1.0. Compared with the free version of Gemini, Gemini Advanced offers more advanced features and a significantly larger context window.

Japanese first-class radiation protection supervisor examination

The Radiation Protection Supervisor is a qualification legally required for supervisors at facilities handling radioactive isotopes or radiation-generating equipment to prevent radiation hazards. This qualification is based on the Act on the Regulation of Radioisotopes, ensuring that the person in charge oversees radiation safety management and assumes legal responsibilities. The examination for certification as a First-Class Radiation Protection Supervisor was conducted by the NRA. The exam is typically held annually in late August in major cities across Japan, such as Sapporo, Tokyo, Osaka, and Fukuoka. The 68th First-Class Radiation Protection Supervisor Examination in Japan was held on August 23 and 24, 2023. The exam covers the following subjects: physics, chemistry, biology, practical operations, and laws and regulations. The exam duration for physics, chemistry, and biology is 110 min. The tests for practical operations and laws and regulations last 100 min and 75 min, respectively. The examination for physics, chemistry, or biology generally comprises 60 multiple-choice questions (30 questions are five options and another 30 questions are several options). One of the 30 multiple-choice questions with five options in physics was excluded due to a misprint in the 68th First-Class Radiation Protection Supervisor Examination. The number of the multiple-choice questions with several options in chemistry was 29 in the exam. Practical operations include 60 multiple-choice questions with several options. Laws and regulations consist of 30 multiple-choice questions with five options. The multiple-choice questions with several options ranged from four to 15 options and consisted of longer passage questions than those with five options. The passing criteria for the exam were scoring 50% or more in each subject and having a total score of 60% or more across all subjects. In the 68th First-Class Radiation Protection Supervisor Examination, the exam had 3,114 candidates, 867 of whom passed, resulting in a pass rate of 27.8% [14]. All authors of this study hold a First-Class Radiation Protection Supervisor license.

Data input

Questions and their multiple-choice answers from the 68th First-Class Radiation Protection Supervisor Examination were used in their original Japanese form. Instructions for using GPT-4 and Gemini Advanced were also provided in Japanese. GPT-4 and Gemini Advanced were able to recognize images, we input both text data and images after the confirmation of understanding the image. The data input was performed in September 2024.

Statistical analysis

Statistical tests were conducted using the chi-square test when the expected values were 5 or more, or the Fisher’s exact test when the expected values were less than 5. A P-value of less than 0.05 was considered statistically significant. Statistical analysis was performed using GraphPad Prism 9.2.0 software (GraphPad Software Inc., USA).

## Results

Both GPT-4 and Gemini Advanced were able to generate answers for all the questions (total 268 questions) in the 68th First-Class Radiation Protection Supervisor Examination posed in Japanese. The results of the 68th First-Class Radiation Protection Supervisor Examination by GPT-4 are shown in Table 1, and those by Gemini Advanced are shown in Table 2.

**Table 1:** The results of the 68th First-Class Radiation Protection Supervisor Examination by GPT-4 The percentage represents the accuracy rate of either correct or incorrect answers. GPT: Generative Pre-trained Transformer, ns: not significant.

Variables	No. of questions	No. of correct answers (%)	No. of incorrect answers (%)	P-value
All questions	268	190 (71.0)	78 (29.0)	
Subject				P < 0.0001
Physics	59	43 (72.9)	16 (27.1)	
Chemistry	59	48 (81.4)	11 (18.6)	
Biology	60	44 (73.3)	16 (26.7)	
Practical operations	60	45 (75.0)	15 (25.0)	
Laws and regulations	30	10 (33.3)	20 (66.7)	
Calculation				ns (P = 0.8966)
Calculation problems	32	23 (71.9)	9 (28.1)	
Non-calculation problems	236	167 (70.8)	69 (29.2)	
Passage length				ns (P = 0.8637)
Short passage questions	149	105 (70.5)	44 (29.5)	
Long passage questions	119	85 (71.4)	34 (28.6)	
Format				P = 0.0003
Textual questions	250	184 (73.6)	66 (26.4)	
Graphical questions	18	6 (33.3)	12 (66.7)	

**Table 2:** The results of the 68th First-Class Radiation Protection Supervisor Examination by Gemini Advanced The percentage represents the accuracy rate of either correct or incorrect answers. GPT: Generative Pre-trained Transformer, ns: not significant.

Variables	No. of questions	No. of correct answers (%)	No. of incorrect answers (%)	P-value
All questions	268	175 (65.3)	93 (34.7)	
Subject				P = 0.0127
Physics	59	38 (64.4)	21 (35.6)	
Chemistry	59	46 (78.0)	13 (22.0)	
Biology	60	39 (65.0)	21 (35.0)	
Practical operations	60	40 (66.7)	20 (33.3)	
Laws and regulations	30	12 (40.0)	18 (60.0)	
Calculation				ns (P = 0.723)
Calculation problems	32	20 (62.5)	12 (37.5)	
Non-calculation problems	236	155 (65.7)	81 (34.3)	
Passage length				ns (P = 0.2673)
Short passage questions	149	93 (62.4)	56 (37.6)	
Long passage questions	119	82 (68.9)	37 (31.1)	
Format				P < 0.0001
Textual questions	250	172 (68.8)	78 (31.2)	
Graphical questions	18	3 (16.7)	15 (83.3)	

Overall accuracy rates were 71.0% and 65.3% in GPT-4 and Gemini Advanced, respectively. A significant difference of the results in the subject was observed in both GPT-4 and Gemini Advanced (P < 0.0001 in GPT-4 and P = 0.0127 in Gemini Advanced). A possible reason for the statistical significance in the subject could be the lower accuracy rate in laws and regulations compared with the other subjects. The multiple-choice questions with several options consisted of longer passage questions than those with five options. Therefore, we classified the questions into two types: short passage questions that typically consisted of one to three sentences (multiple-choice questions with five options) and long passage questions that typically consisted of four or more sentences (multiple-choice questions with several options). In terms of question type, the accuracy rates for calculation problems were 71.9% and 62.5%, for non-calculation problems were 70.8% and 65.7%, for short passage questions were 70.5% and 62.4%, for long passage questions were 71.4% and 68.9%, for textual questions were 73.6% and 68.8%, and for graphical questions were 33.3% and 16.7%, in GPT-4 and Gemini Advanced, respectively. There was no statistically significant difference in the results based on the presence or absence of calculation or the passage length. The performance of both LLMs was significantly better in textual questions than in graphical questions (P = 0.0003 in GPT-4 and P < 0.0001 in Gemini Advanced).

Although the overall accuracy rates in both GPT-4 and Gemini Advanced exceeded 60.0%, which was the passing standard for the average score, the accuracy rates in laws and regulations failed to meet the minimum passing standard of 50%. Therefore, the performance of GPT-4 and Gemini Advanced did not meet the passing criteria if we take into account all the subjects.

Table 3 demonstrates the results of comparative analysis between GPT-4 and Gemini Advanced. There was no statistical significance between GPT-4 and Gemini Advanced in the results of the 68th First-Class Radiation Protection Supervisor Examination.

**Table 3:** The comparison of the 68th First-Class Radiation Protection Supervisor Examination results between GPT-4 and Gemini Advanced GPT: Generative Pre-trained Transformer, ns: not significant.

Variables	No. of correct/incorrect answers		P-value
	GPT-4	Gemini Advanced	
All questions	190/78	175/93	ns (P = 0.1645)
Subject			
Physics	43/16	38/21	ns (P = 0.3211)
Chemistry	48/11	46/13	ns (P = 0.6474)
Biology	44/16	39/21	ns (P = 0.323)
Practical operations	45/15	40/20	ns (P = 0.3153)
Laws and regulations	10/20	12/18	ns (P = 0.5921)
Calculation			
Calculation problems	23/9	20/12	ns (P = 0.4245)
Non-calculation problems	167/69	155/81	ns (P = 0.2355)
Passage length			
Short passage questions	105/44	93/56	ns (P = 0.141)
Long passage questions	85/34	82/37	ns (P = 0.6708)
Format			
Textual questions	184/66	172/78	ns (P = 0.236)
Graphical questions	6/12	3/15	ns (P = 0.443)

## Discussion

LLMs are gaining attention in various fields, including healthcare, for their potential to transform education and research [13,15]. GPT-4 scored high accuracy in radiology board-style examinations, addressing clinical questions in radiology [11,12]. However, it was unclear how well LLMs could perform in the area of radiation safety management. In this study, we assessed the performance of GPT-4 and Gemini Advanced on radiation safety and protection using the 68th First-Class Radiation Protection Supervisor Examination in Japan.

In the Radiation Protection Supervisor Examination, questions related to the basic knowledge of nuclear medicine are asked. Toyama et al. reported that the performance of GPT-4 was significantly better in nuclear medicine (93.3%) than in diagnostic radiology (55.8%) [12]. This is consistent with our results because GPT-4 and Gemini Advanced were able to answer questions well related to radiation physics, chemistry, biology, and practical operations.

Regarding the format, the results were significantly different between textual questions and graphical questions. It has been reported that GPT-4V faces challenges in responding to graphical questions in certain medical fields [16-18]. The current version of GPT-4 also demonstrates an inferior ability to understand and respond to some graphical questions compared with textual questions. Although the number of graphical questions was small in this examination, further training may be required to answer graphical questions in both GPT-4 and Gemini Advanced.

Even though the accuracy rate reached a certain level in several subjects, The accuracy rate of GPT-4 and Gemini Advanced’s answers to questions was not perfect. Hallucinations refer to the phenomenon where LLMs generate false information [19,20]. GPT-4 and Gemini Advanced are designed to minimize hallucinations, but they cannot be completely eliminated. Hallucinations are more likely to occur, especially in response to complex questions or questions about information not included in the training data [19,20]. Therefore, it is important to always critically evaluate responses from LLMs and verify the accuracy of information when necessary.

In this study, there were surprisingly erroneous answers to the questions in laws and regulations. While the training of GPT-4 and Gemini Advanced includes a vast amount of information on laws and regulations, their primary functions are to understand and explain these concepts, rather than to reproduce specific legal texts word-for-word. In addition, the details of laws and regulations are frequently revised. If it becomes necessary to refer to specific legal provisions, it is advisable to review the exact text of laws and regulations and verify their interpretations with First-Class Radiation Protection Supervisors at present.

As of the time of analysis, no significant performance differences were observed between GPT-4 and Gemini Advanced regarding the First-Class Radiation Protection Supervisor Examination. Both LLMs are expected to evolve in the field of radiation safety management.

The 68th First-Class Radiation Protection Supervisor Examination in Japan was performed in Japanese. Japanese often uses indirect expressions and omissions, making it necessary to understand nuances and read between the lines more than in other languages. Therefore, the performance of GPT-4 and Gemini Advanced on the examinations related to Radiation Protection Supervisors in other countries might be different.

## Conclusions

In the Japanese First-Class Radiation Protection Supervisor Examination, the accuracy rates of GPT-4 and Gemini Advanced in answering questions related to physics, chemistry, biology, and practical operations met the passing criteria. However, passing criteria were not met in terms of the accuracy rate for laws and regulations.

Utilizing GPT-4 and Gemini Advanced for radiation medicine education is valuable. At present, when it comes to radiation safety and protection, particularly regarding laws and regulations, it is essential to verify the exact legal text and confirm interpretations with First-Class Radiation Protection Supervisors.
